# West Nile Virus in a changing climate: epidemiology, pathology, advances in diagnosis and treatment, vaccine designing and control strategies, emerging public health challenges – a comprehensive review

**DOI:** 10.1080/22221751.2024.2437244

**Published:** 2024-11-30

**Authors:** Parminder Singh, Mahalaqua Nazli Khatib, Suhas Ballal, Mandeep Kaur, Deepak Nathiya, Shilpa Sharma, G. V. Siva Prasad, Aashna Sinha, Abhay M. Gaidhane, Priyanka Mohapatra, Amit Varma, Sorabh Lakhanpal, Muhammed Shabil, Ganesh Bushi, Sanjit Sah, Hashem Abu Serhan

**Affiliations:** a Center for Global Health Research, Saveetha Medical College and Hospital, Saveetha Institute of Medical and Technical Sciences, Saveetha University, Chennai, India; b Division of Evidence Synthesis, Global Consortium of Public Health and Research, Datta Meghe Institute of Higher Education, Wardha, India; c Department of Chemistry and Biochemistry, School of Sciences, JAIN (Deemed to be University), Bangalore, India; d Department of Allied Healthcare and Sciences, Vivekananda Global University, Jaipur, India; e Department of Pharmacy Practice, Institute of Pharmacy, NIMS University, Jaipur, India; f Chandigarh Pharmacy College, Chandigarh Group of Colleges-Jhanjeri, Mohali, India; g Department of Chemistry, Raghu Engineering College, Visakhapatnam, India; h Division of Research and Innovation, Uttaranchal Institute of Pharmaceutical Sciences, Uttaranchal University, Dehradun, India; i Jawaharlal Nehru Medical College, and Global Health Academy, School of Epidemiology and Public Health, Datta Meghe Institute of Higher Education, Wardha, India; j Evidence for Policy and Learning, Global Center for Evidence Synthesis, Chandigarh, India; k Department of General Medicine, Graphic Era (Deemed to be University), Dehradun, India; l School of Pharmaceutical Sciences, Lovely Professional University, Phagwara, India; m University Center for Research and Development, Chandigarh University, Mohali, India; n Medical Laboratories Techniques Department, AL-Mustaqbal University, Babil, Iraq; o Noida Institute of Engineering and Technology (Pharmacy Institute), Greater Noida, India; p Department of Paediatrics, Dr. D. Y. Patil Medical College, Hospital and Research Centre, Pune, India; q Department of Public Health Dentistry, Dr. D.Y. Patil Dental College and Hospital, Pune, India; r Department of Medicine, SR Sanjeevani Hospital, Kalyanpur, Siraha, Nepal; s Department of Ophthalmology, Hamad Medical Corporation, Doha, Qatar

**Keywords:** West Nile Virus, epidemiology, neuroinvasiveness, vector control, and vaccine development

## Abstract

West Nile Virus (WNV), first identified in Uganda in 1937, remains a significant global health threat, adapting across diverse ecosystems and expanding geographically, particularly into temperate regions of Europe and North America. This review provides a comprehensive exploration of the latest insights and challenges in WNV management, focusing on epidemiological trends, molecular advancements, and public health implications. Recent data highlight WNV's expansion, driven by climate changes such as milder winters and longer warm seasons that increase mosquito activity and enable the virus to overwinter within mosquito populations. This facilitates year-round transmission and challenges current control strategies. Molecularly, advancements in genomic and proteomic technologies have deepened our understanding of WNV's replication and pathogenesis, identifying new therapeutic targets and improving diagnostic methods. However, the absence of an approved human vaccine leaves management dependent on supportive care, particularly for severe neurological cases. Effective vector control remains crucial, with innovative strategies including genetically modified mosquitoes and novel insecticides being pivotal. Furthermore, environmental factors like climate change and urbanization are altering vector behaviors and WNV transmission dynamics, necessitating adaptive public health strategies to manage these evolving threats. The review underscores the need for ongoing research, vaccine and therapeutic development, and enhanced public health infrastructures to better respond to WNV challenges. It stresses the critical role of integrating scientific research, public health policy, and community engagement to effectively address the persistent threat of WNV.

## Introduction

West Nile Virus (WNV), a mosquito-borne neurotropic virus first identified in the West Nile region of Uganda in 1937 [1], belongs to the Flaviviridae family and the Orthoflavivirus genus [2]. As a member of the Japanese encephalitis serocomplex [3], WNV has established itself as a significant global health threat, adapting to a variety of ecological settings and spreading across continents. Virus resistance and geographic dissemination are determined by complex interactions among environmental alterations, host populations, and vector dynamics, rendering it a crucial area of investigation for emerging infectious illnesses affected by global changes [1,4]. Epidemiological trends have shown a notable expansion in WNV's range, with increases in incidence and outbreak severity particularly observed in Europe and North America [5]. These changes are largely driven by climatic factors such as milder winters and extended warm seasons, which enhance mosquito activity and prolong transmission periods [6]. Research indicates that WNV has adapted to overwinter in mosquito populations in Europe, suggesting the potential for year-round transmission in some temperate areas, a shift that poses significant challenges to current control strategies [7]. On the molecular level, advancements in genomic and proteomic technologies have deepened our understanding of WNV's replication and pathogenic mechanisms, uncovering new therapeutic targets [8]. The exploration of WNV's genetic diversity and evolutionary dynamics through next-generation sequencing offers valuable insights into its adaptability and the factors influencing its spread [9].

In the realm of public health, there is no approved vaccine for WNV in humans, though several candidates are undergoing clinical trials [10]. Management of severe WNV cases, particularly those involving neurological symptoms, remains largely supportive, with ongoing research into antiviral treatments that target specific stages of the viral life cycle [11]. Vector control remains a cornerstone of WNV management, relying heavily on controlling mosquito populations. Innovations such as genetically modified mosquitoes and novel insecticidal approaches show promise in reducing transmission rates [12]. Enhanced surveillance systems that monitor mosquito and bird populations help predict outbreaks and inform public health responses [13]. In order to handle these complex issues, this emphasises the significance of a One Health strategy that integrates human, animal, and environmental health concepts [14]. However, emerging challenges such as climate change continue to alter the behavior of mosquito vectors and the dynamics of WNV transmission [15]. For example, warmer temperatures can lead to more frequent and intense heatwaves, which accelerate mosquito breeding cycles and expand their geographic range. Urbanization and land-use changes further complicate the ecological balance, influencing vector populations and virus spread [6]. Increased urban density, for instance, often results in more stagnant water bodies through poor water management, providing ideal breeding grounds for mosquitoes. Adapting public health strategies to these evolving conditions is essential for effective WNV management.

Future efforts must focus on continuous research to refine our understanding of WNV, the development of effective vaccines and therapeutics, and the strengthening of public health infrastructures capable of rapid response. International collaboration will be crucial in addressing the global challenge posed by WNV as it continues to expand its reach into new regions and reemerge in established ones. This comprehensive approach highlights the complex interplay between scientific research, public health policy, and community engagement in combating this enduring health threat.

## 3D structure of West Nile Virus and its role in disease mechanisms

**Figure 1.**
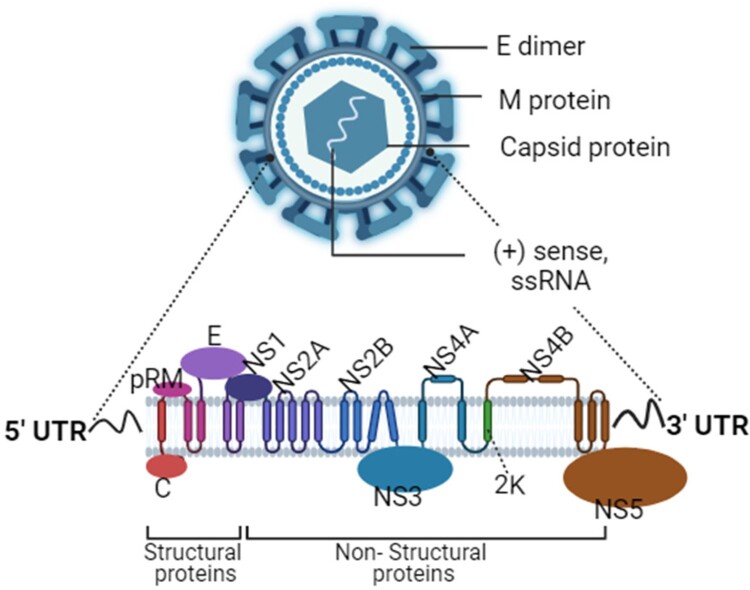
Structural organization of the west nile virus genome.

The three-dimensional structure of the West Nile virus (WNV) is crucial for understanding its pathogenesis and interaction with host cells. Figure 1 presents a thorough overview of the West Nile Virus genome, depicting both its structural and non-structural proteins. The West Nile virus genome encodes a polyprotein from a single open reading frame (ORF), which is cleaved co- and post-translationally by host and viral proteases. This results in the formation of structural proteins, including the Capsid (C), pre-Membrane (prM), and Envelope (E) proteins, which are essential for virus assembly and entry into host cells [15]. Additionally, several non-structural proteins, such as NS1, NS2A, NS2B, NS3, NS4A, NS4B, and NS5, are produced, playing crucial roles in viral replication and immune evasion [16]. The envelope glycoprotein (E) of WNV, critical for viral entry, undergoes a conformational rearrangement triggered by the low pH within the endosome, resulting in a class II fusion event. The crystal structure of the ectodomain of WNV E reveals a three-domain architecture shared with other flaviviruses like dengue and tick-borne encephalitis viruses. Notably, the WNV E protein crystallized as a monomer, unlike other flavivirus E proteins that form antiparallel dimers [17]. However, while the E protein is depicted as a dimer in the Figure 1 (reflecting its biological role in virion formation), under specific crystallization conditions it appears as a monomer. This unique monomeric form assembles in a crystalline lattice of perpendicular molecules, with the fusion loop of one E protein buried in a hydrophobic pocket at the DI-DIII interface of another, potentially allowing virion conformational transitions while minimizing fusion loop exposure [17]. The pathogenesis of WNV is also influenced by its interactions with human proteins. A network of 3,346 interactions involving 6 WNV proteins and 1,970 human target proteins has been constructed, revealing that these human proteins tend to be central and conserved within the human protein–protein interaction (PPI) network [18]. These target proteins are mainly involved in processes such as virus process, transcription regulation, and cell adhesion, which are critical for viral replication and immune response evasion. Additionally, a single amino acid change in the NS3 helicase of North American WNV isolates has been associated with increased virulence in certain avian species, which has implications for the virus's spread and intensity of transmission [19].

## Pathogenesis and neurotropic characteristics

**Figure 2.**
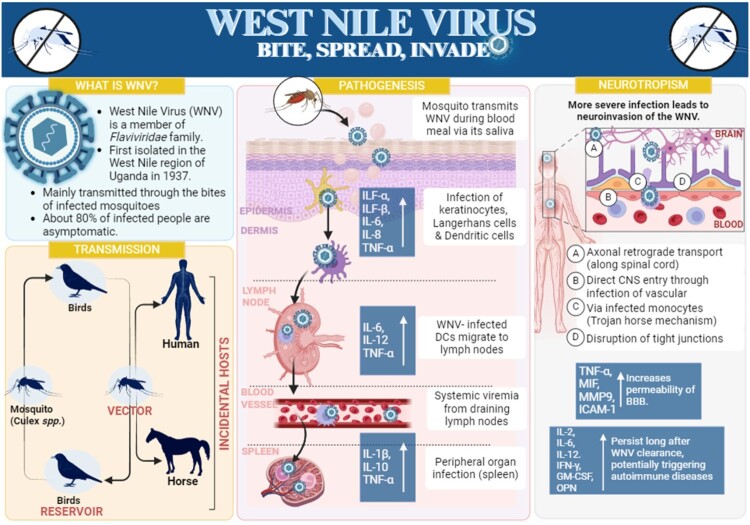
Comprehensive overview of West Nile Virus transmission, pathogenesis, and neurotropism.

The West Nile Virus presents considerable public health issues owing to its pathogenesis and neurotropic properties. The virus is primarily transmitted by Culex mosquitoes [16], utilizing birds as its main reservoir, while humans and other animals serve as incidental hosts (Figure 2 (Transmission panel)) [20]. The first infection transpires at the epidermal layer, where the virus infiltrates via mosquito bites, mostly affecting keratinocytes before progressing to dendritic cells. These cells facilitate viral dissemination to lymph nodes, prompting replication and viremia, which enable further spread to various organs and potentially crossing the blood–brain barrier (BBB) (Figure 2) (Pathogenesis Panel) [21]. The neuroinvasiveness of WNV is particularly alarming, characterized by its ability to infect neural cells, an attribute mediated by the viral envelope protein that binds to cellular receptors. Once inside the central nervous system (CNS), WNV induces neuroinflammation and neuronal death, contributing to severe clinical manifestations such as meningitis, encephalitis, and acute flaccid paralysis [22]. These conditions underscore the dual role of the immune response; it is pivotal in controlling the infection and, paradoxically, it exacerbates neurological damage [20, 23].

Neurotropism, a hallmark of WNV, leads to serious neurological consequences and substantial morbidity (Figure 2) (Neurotropism Panel) [24]. The mechanisms underlying this feature include the direct viral infection of endothelial cells of the BBB and the Trojan horse mechanism, where the virus hijacks immune cells to cross the BBB [25]. The resulting inflammatory response within the CNS can disrupt neural functions and exacerbate the disease's severity [26]. Research into WNV's interaction with the host's immune system is crucial for developing targeted therapies and vaccines, particularly those focusing on the viral envelope protein and its replication processes. Public health efforts to control mosquito populations and prevent bites are essential in mitigating WNV transmission. The comprehensive understanding of WNV pathogenesis and neurotropism not only aids in managing this specific virus but also enhances our readiness against similar neurotropic pathogens [8].

## Recent epidemiological trends

West Nile Virus, has shown evolving epidemiological trends, with recent data indicating significant changes in the virus geographic spread and outbreak intensity. In Greece, a study documented the overwintering of WNV in active mosquito populations during the winter of 2022, suggesting potential year-round circulation in some European regions [4]. This finding underscores the need for extended surveillance programs in temperate climates and historically affected areas. In Europe, the geographic reach of WNV has expanded northward, with initial human cases reported in Germany and the Netherlands in recent years [5]. This expansion is driven by favorable temperature conditions for mosquito vectors. Mathematical modeling across five European countries shows that warmer temperatures have led to more transmission days, particularly in southern Europe [7]. Similarly, in the United States, a study noted broadscale spatial synchrony in mosquito populations, influenced by climatic factors such as temperature and precipitation [27]. This synchrony is crucial for understanding WNV transmission dynamics, especially given the projected increase in temperature, which is expected to escalate future WNV cases in regions like California. An unusual early surge of WNV cases in Israel in June 2024, including a high number of neuroinvasive diseases, highlights the potential impact of early seasonal temperature rises on virus transmission [28]. In Serbia, spring temperatures have been identified as a key factor influencing the timing and magnitude of WNV outbreaks [29].

Climate change emerges as a significant driver of WNV transmission dynamics, with warmer winters and springs linked to more intense virus circulation, particularly noted in southern Spain [30]. These climatic changes facilitate the expansion of mosquito vectors, which in turn enhances virus transmission. This connection suggests that temperature could serve as an early warning signal for intensifying WNV activity. The recent epidemiological data underscore the challenges posed by WNV, particularly highlighting how climatic factors, including rising temperatures and vector habitat expansion, influence its transmission [31]. The absence of effective vaccines and specific treatments for WNV makes prevention strategies focused on mosquito control and surveillance crucial. Moreover, the geographical spread of vectors due to climate change necessitates proactive strategies to mitigate the impact of this virus on public health [21].

Recent epidemiological data have provided new insights into West Nile Virus (WNV) transmission dynamics, emphasizing the adaptability of the virus to changing climatic conditions and urban landscapes. This adaptability has facilitated the virus's persistence and spread into new geographical areas, presenting challenges that require dynamic and informed public health responses. Notably, the identification of overwintering WNV in European mosquito populations indicates potential for year-round transmission cycles, underscoring the urgency for enhanced surveillance and tailored intervention strategies. Integrated approaches, such as the One Health strategy, which consider the interconnection between people, animals, and their shared environment, are essential [14]. These findings mark a critical evolution in our understanding of WNV epidemiology, distinctly shaping the ongoing development of global health strategies.

## Phylogenetics analysis

**Figure 3.**
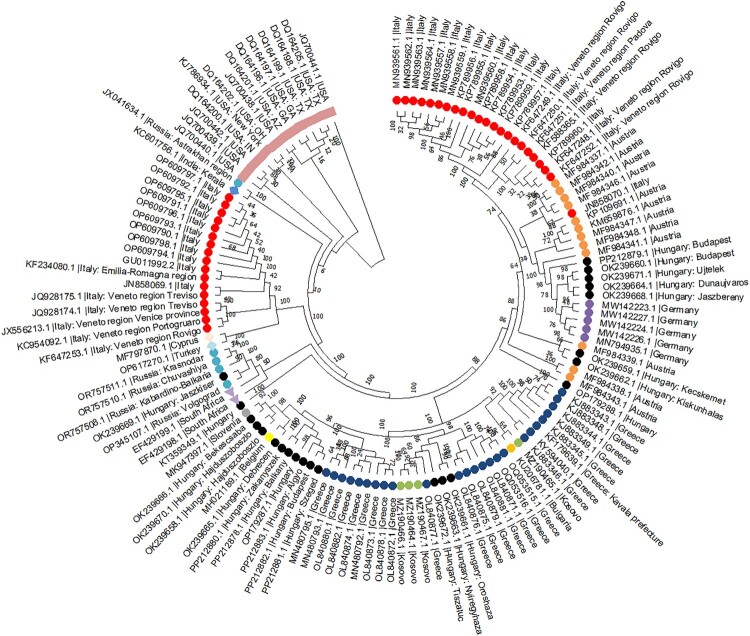
Phylogenetic analysis of West Nile Virus: global spread and diversification.

The West Nile virus nucleotide sequence was obtained for a pairwise similarity analysis using the NCBI virus taxonomy database. Based on their entire nucleotide sequence and host homo-sapiens, we selected 136 nucleotide sequences out of 23,303 nucleotide sequences for examination. The sequence alignment was finished by using the MUSCLE tool using the default settings included in MEGA11. Using the Maximum Likelihood technique (50 Bootstrap replication) and the Tamura-Nei model as a replacement model, phylogenetic analysis was performed in Mega11 [32,33]. The tree with the highest log likelihood (−51055.56) is shown in Figure 3.

The phylogenetic tree provided offers a detailed visual representation of the genetic relationships and diversification of WNV strains across different global regions, as indicated by the specific markers and colors for each country. This tree is crucial for understanding the evolutionary pathways and migration patterns of the virus, shedding light on its spread and the emergence of different lineages over time. European Strains (Circle Shape) Predominantly clustered in the upper and middle sections of the tree, these strains demonstrate a considerable diversity within Europe itself, as evidenced by the wide distribution across the tree. The colors representing different countries within Europe help to illustrate the specific regions where distinct strains have been identified, with a high concentration in Italy, Greece, and Hungary shown in red, blue, and black. This suggests active circulation and possibly continuous evolution of the virus within these regions. North American Strains (Square Shape) are relatively less diverse in this tree, indicating either a more recent introduction or less genetic variability among the strains sampled from the USA. The placement of these strains within the tree suggests possible introductions from Europe, as they are interspersed among European strains. Asian Strains (Diamond Shape) are represented by only a few strains in this tree, the Asian variants are closely related to some of the European strains, particularly those from Russia, suggesting a possible migration route or common ancestry between these regions. African Strains (Triangle Shape) are also represented by only two strains and variants are closely related to the Russian strain. Understanding the phylogenetic relationships helps in tracing the transmission pathways and can aid in predicting future spread patterns. The presence of closely related strains across different continents underscores the role of global travel and bird migration in disseminating the virus.

## Advances in diagnosis

The diagnosis of West Nile Virus has evolved significantly with the adoption of advanced molecular diagnostics and real-time PCR techniques. These enhancements have notably improved the sensitivity, specificity, and efficiency of detecting this potentially life-threatening infection. Notably, the introduction of urine quantitative RT–PCR (qRT-PCR) has emerged as a valuable tool, particularly for confirming WNV neuroinvasive disease [34]. In comparison to blood testing, this approach has been demonstrated to detect the virus in urine at higher concentrations and over longer periods of time. This has resulted in notable improvements in case confirmation rates, with 31.7% more cases identified than with serum testing alone [34]. Furthermore, the utilization of whole blood for PCR testing, as evidenced during the 2021 Arizona outbreak, has proven more effective than cerebrospinal fluid (CSF) or serum testing alone [35]. This method has enabled more comprehensive detection and provided timely results, which are critical for the effective management of neuroinvasive disease. Despite the pivotal role of PCR, serological testing remains crucial. Innovations in ELISA techniques have enhanced the detection of WNV-specific IgM and IgG antibodies, facilitating earlier and more reliable diagnoses. However, despite improvements, particularly with NS1-based ELISAs, challenges in reducing cross-reactivity with other flaviviruses persist, especially in regions with multiple endemic flaviviruses and prevalent flavivirus vaccinations [36].

Additionally, new biomarkers like Platelet Distribution Width (PDW) have been identified, which could further refine the diagnostic process, offering high sensitivity and specificity in distinguishing WNV infections from other febrile illnesses [37]. The advent of point-of-care (POC) tests, such as the loop-mediated isothermal amplification (LAMP) assay, represents a revolution in WNV diagnostics, especially beneficial in resource-limited settings [38]. These rapid, accurate, and portable tests provide critical advantages during outbreak situations, facilitating immediate decision-making and action. Advanced modeling techniques using environmental and anthropic variables have also been employed to predict high-risk areas for WNV transmission. Such predictive models have proven effective in places like the Iberian Peninsula, aiding preemptive public health interventions by forecasting potential human and equine cases [39]. Despite these advancements, challenges remain, particularly in differentiating WNV from other viral infections due to similar clinical presentations. The integration of specific CSF parameters and demographic data has shown promise in improving diagnostic accuracy for WNV, particularly for neuroinvasive cases [40].

## Advances in treatment

The battle against West Nile Virus has seen significant advancements in treatment strategies, particularly through novel antiviral agents and innovative immunotherapeutic approaches, even as the search for an effective vaccine continues. Research has increasingly focused on targeting specific viral components crucial for WNV replication, highlighting the potential of specific inhibitors. For example, studies have identified non-nucleoside inhibitors that target the RNA-dependent RNA polymerase (RdRp), an enzyme pivotal for the viral replication cycle, promising specificity in targeting WNV while minimizing off-target effects on host cells [41]. The strategy of repurposing existing clinically approved drugs for WNV treatment offers a rapid avenue to identify effective therapies, given their established safety profiles. Research has screened existing drugs to identify several compounds with potent anti-WNV activity, which accelerates the therapeutic pipeline by leveraging drugs that are already available on the market [42].

In the realm of immunotherapy, strategies have shown promise by enhancing the body's immune response to WNV. Innovative work has led to the development of neutralizing antibodies that not only target WNV but also offer cross-protection against related viruses, such as the Japanese encephalitis virus [43]. This dual-function capability is particularly valuable in regions where multiple flaviviruses are endemic, suggesting a broad application for these immunotherapeutic developments. Further, understanding the interaction between WNV and host factors through advanced genetic and molecular techniques provides critical insights necessary for developing targeted therapies. For instance, research into how host protein CD11b influences WNV replication and modulates the immune response could lead to targeted treatments that effectively modulate host-virus interactions [44]. For individuals already infected with WNV, especially those with severe manifestations like neuroinvasive disease, supportive care remains a critical component of treatment [22]. Investigations into the impact of steroid therapy on outcomes in West Nile encephalitis have been conducted, though more research is needed to establish definitive treatment protocols [23].

Overall, the advancement in the treatment of WNV encompasses a spectrum from molecular targeted therapies and repurposed drugs to innovative immunotherapies. These developments not only offer hope for more effective management of WNV but also underscore the importance of an integrated approach that combines therapeutic innovations with preventive strategies. Continued research and collaboration across disciplines are vital to transform these scientific insights into practical solutions that can alleviate the global burden of West Nile Virus.

## Vector control and prevention strategies

West Nile virus poses significant health risks to humans and animals as a mosquito-borne pathogen, necessitating effective control and prevention strategies. Advances in molecular techniques, such as DNA sequencing to determine mosquito feeding patterns, have enhanced our understanding of vector behavior, aiding in targeted control efforts that reduce mosquito populations in crucial areas [9]. Concurrently, the exploration of botanical pesticides offers an eco-friendly alternative to traditional chemical pesticides. Studies have shown that essential oils like lavender, peppermint, and rosemary effectively control mosquito populations by acting as larvicidal and adulticidal agents [13]. Moreover, genetically modified mosquitoes that have reduced vector competence show promise in controlling disease transmission [11]. However, progress in this area has predominantly been with Aedes species, which are not the primary vectors for WNV [45]. For Culex species, which are significant in WNV transmission, ongoing research and developments are required to establish similarly effective vector control solutions with minimal ecological impact.

Integrated Vector Management (IVM) combines physical, chemical, biological, and environmental techniques to manage mosquito populations effectively, particularly in urban areas where vectors are prevalent [12]. Public education and community engagement also play crucial roles in vector control strategies. By raising awareness about WNV and promoting actions to eliminate mosquito breeding sites, such initiatives can significantly reduce local mosquito populations. However, challenges such as insecticide resistance and environmental changes like climate change and urbanization complicate the control of WNV [46]. Future research should focus on developing sustainable vector control methods and vaccines, particularly for humans, to address these challenges effectively. Overall, a multi-faceted approach involving advanced molecular techniques, genetic engineering, integrated management strategies, and community involvement represents the best strategy for mitigating the public health threat posed by WNV [11,12].

## Progress in vaccine development

West Nile virus is a significant public health concern due to its potential to cause severe neurological conditions and even death. Despite extensive efforts, there is no commercially available human vaccine for WNV, and control measures primarily focus on vector management and personal protection [47]. Remarkable success has also been achieved with horse vaccinations, where multiple licensed vaccines such as West Nile-Innovator by Zoetis and Recombitek Equine West Nile Virus vaccine by Merial have effectively controlled WNV in equine populations, demonstrating a model for potential human vaccine strategies [48,49]. However, recent advancements in vaccine research offer hope for effective prophylactic solutions against this virus. Recent studies have explored various approaches for WNV vaccine development. One promising avenue is the use of inactivated vaccines, as demonstrated in a field study where zoo birds were vaccinated against WNV using a commercial inactivated vaccine [50]. This study reported a significant increase in neutralizing antibody titers, particularly in birds that received the highest dose of the vaccine, suggesting the potential efficacy of inactivated vaccines in providing immunity against WNV. Another innovative approach involves the use of chimeric vaccines. For example, a study tested a chimeric vaccine combining the non-structural genes of the insect-specific flavivirus Binjari with the structural proteins of WNV [51]. This vaccine was shown to be effective in preventing WNV-induced skin lesions in farmed crocodiles, highlighting the potential of chimeric vaccines to provide cross-species protection. Table 1 presents the salient features of clinical trials pertaining to several West Nile virus vaccines, as gathered from clinicaltrails.gov. It emphasises the vaccines’ safety, potential side effects, and immunogenicity in relation to different age groups and dosages [52].

**Table 1.** Overview of clinical trials and observations for West Nile Virus vaccines: safety, adverse effects, and immunogenicity.

Vaccine Name	Vaccine Type	Clinical Trial ID	Age Group	Dose	Adverse Effects	Immunogenicity Observations
ChimeriVax-WN02	Live attenuated chimeric	NCT00442169	Part1 18–40 Part2 41–64 and ≥ 65 years	Part1 3.7 × – 10(5) PFU, 3.7 ×10(4) PFU, 3.7 × 10(3) PFU Part2 3.7 × 10(5) PFU	Similar adverse event profile across all dose groups; more events in 41–64 years cohort.	High seroconversion rates; higher antibody titers and lower viremia levels with the highest dose.
WN/DEN4delta30	Live attenuated	NCT00537147	Adults (18–50 years old)	Experiment1 10^4^ PFU (0.5 ml) Experiment2 10^5^ PFU (0.5 ml)	Vaccine-related adverse events classified by both intensity and severity.	Immunogenicity assessed by anti-WN/DEN4 neutralizing antibody titer.
WN-80E	Protein-based	NCT00707642	Adults (18–45 years old)	Experiment1 5 µg + Alhydrogel (3.5 mg) Experiment 2 15 µg + Alhydrogel (3.5 mg) Experiment 3 50 µg + Alhydrogel (3.5 mg) Experiment4 50 µg + no adjuvant	No serious adverse biological effects reported.	Immunogenic in Nene; high antibody titers indicating good immunogenicity.
HydroVax-001 WNV	Inactivated, whole virion	NCT02337868	Adults 18–50 years	1 and 4 mcg	Well-tolerated; no serious adverse events. Increased reactogenicity with complement.	Modest immunogenicity at 4 mcg dose; higher PRNT(50) seroconversion rates with added complement.
VRC-WNVDNA020-00-VP	DNA vaccine	NCT00300417	Adults (18–65 year old)	Not specified	Participants reported symptoms like fever, headache, neck stiffness, muscle weakness, vision loss, numbness, and paralysis, monitored via diary cards.	Vaccine-induced immunity assessed via blood tests for immune response; details not specified.
VRC-WNVDNA017-00-VP	DNA vaccine	NCT00106769	Adults 18–50 years	Not specified	Participants reported experiencing fever, headache, and muscle weakness via diary cards after vaccination.	Generated neutralizing antibody and T cell responses; protective in studies of incidental natural host for WNV

Technological advancements have also facilitated the development of vaccine adjuvants to enhance immune responses. A study investigated the delivery of small molecule mast cell activators (MCAs) using acetalated dextran microparticles as a novel adjuvant strategy [53]. This method enhanced the immunogenicity of the West Nile Virus Envelope III protein, demonstrating a potential pathway to improve vaccine efficacy through innovative adjuvant systems. Immunoinformatics is also playing a crucial role in identifying potential vaccine candidates. A recent study employed immunoinformatics tools to identify immunodominant epitopes from the WNV proteome, aiming to design a multi-peptide vaccine that elicits broad humoral and cellular immune responses [54]. This approach highlights the utility of computational methods in accelerating vaccine development by pinpointing effective components of the virus that can be targeted by the immune system⁣. Additionally, research into live-attenuated vaccines continues to show promise. A novel vaccine strategy involving large replacement of the 3’ UTR with an internal poly(A) tract in the WNV genome has shown to attenuate the virus while maintaining immunogenicity in a mouse model [55]. This approach could provide a safe and effective method for developing live-attenuated vaccines for flaviviruses, including WNV.

A blend of conventional and cutting-edge methods is helping to advance the creation of vaccinations against the West Nile virus. With the development of novel adjuvant technologies, inactivated vaccines, and state-of-the-art immunoinformatics techniques, scientists are making great progress in developing potent preventive measures against this perilous virus. There is optimism that these vaccine candidates, as they move through clinical trials, will eventually result in a licensed vaccine that will protect populations at risk of WNV infection.

## Emerging public health challenges

West Nile virus (WNV) continues to pose significant public health challenges due to its ability to cause severe disease and its extensive geographical distribution. One of the critical issues in controlling WNV transmission is the development of resistance to traditional insecticides, particularly in Culex pipiens, the primary vector [4]. This has spurred research into alternative solutions, such as cationic gemini surfactants, which show promise in combating these resistant mosquito populations [56]. Additionally, the changing global climate exacerbates WNV control efforts, as rising temperatures and shifting precipitation patterns expand mosquito habitats, increasing the potential for WNV outbreaks in previously unaffected regions [21]. This highlights the need for robust vector surveillance and rapid response systems to effectively manage outbreaks as they arise. Advances in diagnostic tools, such as the loop-mediated isothermal amplification (LAMP) assay, have improved the speed and accuracy of WNV detection, which is crucial for timely intervention and outbreak containment [38]. However, while veterinary vaccines have been developed to protect animals, the lack of an approved human vaccine underscores the urgent need for further research into safe and effective preventive measures for humans [51].

Moreover, WNV infection can lead to long-term neurological complications in survivors, such as memory loss, tremors, and fatigue [57]. Addressing these chronic conditions requires not only initial acute care but also long-term healthcare strategies to improve survivors’ quality of life. Therefore, effective public health management of WNV must adopt a comprehensive approach that integrates preventive strategies, rapid outbreak response, and sustained support for those affected by long-term health consequences [58]. This multifaceted strategy, combining innovations in medical research, environmental management, and patient care, is essential to mitigating the public health impact of WNV.

## Future outlook

West Nile virus (WNV) continues to challenge public health systems, yet advancements in research and technology offer promising pathways for better management and prediction. Vaccine development is accelerating with innovative candidates like chimeric and multi-peptide vaccines being explored through immunoinformatics to identify immunodominant epitopes for more precise antigen targeting [59]. Concurrent advancements in antiviral therapies, such as the discovery of inhibitors targeting the viral RNA-dependent RNA polymerase via high-throughput screening, pave the way for therapeutics that could lessen the severity of WNV infections [41]. In vector control, novel larvicides like cationic gemini surfactants and eco-friendly methods such as botanical insecticides and genetically modified mosquitoes present sustainable alternatives to traditional methods, which are often hindered by resistance.

Next-generation sequencing (NGS) has significantly enhanced our understanding of mosquito feeding patterns, moving beyond traditional serological and molecular techniques that provided limited resolution and speed. NGS offers a comprehensive, high-resolution analysis of blood meal sources, enabling more precise mapping of WNV transmission networks [9]. This method is particularly valuable in complex ecological settings where multiple potential hosts coexist, allowing for targeted vector control interventions that are both effective and environmentally conscious. Additionally, predictive models integrating environmental, meteorological, and biological data, along with tools like real-time RT-LAMP assays and geospatial mapping, are revolutionizing our ability to forecast and respond to WNV outbreaks swiftly and accurately [38].

## Data Availability

The data is with the authors and available on request.

## References

[1] Chowdhury P, Khan SA. Global emergence of West Nile virus: threat & preparedness in special perspective to India. Indian J Med Res. 2021;154(1):36-50. doi:10.4103/ijmr.IJMR_642_19 34782529 PMC8715705

[2] Terrell JR, Le TT, Paul A, Brinton MA, Wilson WD, Poon GMK, et al. Structure of an RNA G-quadruplex from the West Nile virus genome. Nat Commun. 2024;15(1):5428. doi:10.1038/s41467-024-49761-5 38926367 PMC11208454

[3] Khare B, Kuhn RJ. The Japanese encephalitis antigenic complex viruses: from structure to immunity. Viruses. 2022;14(10):2213. 36298768 10.3390/v14102213 PMC9607441

[4] Balatsos G, Beleri S, Tegos N, Bisia M, Karras V, Zavitsanou E, et al. Overwintering West Nile virus in active Culex pipiens mosquito populations in Greece. Parasit Vectors. 2024;17(1):286. doi:10.1186/s13071-024-06367-6 38956733 PMC11221078

[5] Erazo D, Grant L, Ghisbain G, Marini G, Colon-Gonzalez FJ, Wint W, et al. Contribution of climate change to the spatial expansion of West Nile virus in Europe. Nat Commun. 2024;15(1):1196. doi:10.1038/s41467-024-52036-8 38331945 PMC10853512

[6] Flores-Ferrer A, Suzan G, Waleckx E, Gourbiere S. Assessing the risk of West Nile Virus seasonal outbreaks and its vector control in an urbanizing bird community: An integrative R0-modelling study in the city of Merida, Mexico. PLoS Negl Trop Dis. 2023;17(5):e0011340. 37253060 10.1371/journal.pntd.0011340 PMC10256229

[7] de Freitas Costa E, Streng K, de Souza Santos A, et al. The effect of temperature on the boundary conditions of West Nile virus circulation in Europe. PLoS Negl Trop Dis. 2024;18(5):e0012162. 38709836 10.1371/journal.pntd.0012162 PMC11098507

[8] Akash S, Bayil I, Rahman MA, Mukerjee N, Maitra S, Islam MR, et al. Target specific inhibition of West Nile virus envelope glycoprotein and methyltransferase using phytocompounds: an in silico strategy leveraging molecular docking and dynamics simulation. Front Microbiol. 2023;14:1189786. doi:10.3389/fmicb.2023.1189786 37455711 PMC10338848

[9] Abbasi I, Akad F, Studentsky L, et al. A next-generation (DNA) sequencing (NGS)-based method for identifying the sources of sugar meals in mosquito vectors of West Nile virus in Israel. J Vector Ecol. 2022;47(1):109–116. doi:10.52707/1081-1710-47.1.109 36629362

[10] Carolyn VG, Staples JE, Huang Claire YH, et al. Combating West Nile Virus disease — time to revisit vaccination. N Engl J Med. 2023;388(18):1633–1636. doi:10.1056/NEJMp2301816 37125778 PMC11627013

[11] Cendejas PM, Goodman AG. Vaccination and control methods of West Nile Virus infection in equids and humans. Vaccines (Basel). 2024;12(5):485. 38793736 10.3390/vaccines12050485 PMC11125624

[12] Coatsworth H, Lippi CA, Vasquez C, et al. A molecular surveillance-guided vector control response to concurrent dengue and West Nile virus outbreaks in a COVID-19 hotspot of Florida. Lancet Reg Health Am. 2022;11:100231. 36778921 10.1016/j.lana.2022.100231 PMC9903724

[13] El-Kasem Bosly HA. Larvicidal and adulticidal activity of essential oils from plants of the Lamiaceae family against the West Nile virus vector, Culex pipiens (Diptera: Culicidae). Saudi J Biol Sci. 2022;29(8):103350. doi:10.1016/j.sjbs.2022.103350 35762012 PMC9232543

[14] Agrawal KSS, Singh V, Rohilla R, et al. One health concepts and its applications in clinical practice: a comprehensive review. Evidence. 2024:2:1–10.

[15] Habarugira G, Suen WW, Hobson-Peters J, et al. West Nile Virus: an update on pathobiology, epidemiology, diagnostics, control and “One Health” implications. Pathogens. 2020;9(7):589. doi:10.3390/pathogens9070589 32707644 PMC7400489

[16] Saiz JC, Martin-Acebes MA, Blazquez AB, et al. Pathogenicity and virulence of West Nile virus revisited eight decades after its first isolation. Virulence. 2021;12(1):1145–1173. doi:10.1080/21505594.2021.1908740 33843445 PMC8043182

[17] Nybakken GE, Nelson CA, Chen BR, et al. Crystal structure of the West Nile virus envelope glycoprotein. J Virol. 2006;80(23):11467–11474. doi:10.1128/JVI.01125-06 16987985 PMC1642602

[18] Chen J, Sun J, Liu X, et al. Structure-based prediction of West Nile virus-human protein-protein interactions. J Biomol Struct Dyn. 2019;37(9):2310–2321. doi:10.1080/07391102.2018.1479659 30044201

[19] Diamond MS. Virus and host determinants of West Nile virus pathogenesis. PLoS Pathog. 2009;5(6):e1000452. doi:10.1371/journal.ppat.1000452 19557199 PMC2695557

[20] Ferraguti M, Dimas Martins A, Artzy-Randrup Y. Quantifying the invasion risk of West Nile virus: insights from a multi-vector and multi-host SEIR model. One Health. 2023;17:100638. doi:10.1016/j.onehlt.2023.100638 38024254 PMC10665159

[21] Frasca F, Sorrentino L, Fracella M, et al. An update on the entomology, virology, pathogenesis, and epidemiology status of West Nile and dengue viruses in Europe (2018-2023). Trop Med Infect Dis. 2024;9(7):166. 39058208 10.3390/tropicalmed9070166 PMC11281579

[22] Roberts JA, Kim CY, Dean A, et al. Clinical and diagnostic features of West Nile virus neuroinvasive disease in New York City. Pathogens. 2024;13(5):382. doi:10.3390/pathogens13050382 38787234 PMC11123700

[23] Colaneri M, Lissandrin R, Calia M, et al. The WEST study: a retrospective and multicentric study on the impact of steroid therapy in West Nile encephalitis. Open Forum Infect Dis. 2023;10(3):ofad092. doi:10.1093/ofid/ofad092 36949874 PMC10026538

[24] Shahhosseini N, Moosa-Kazemi SH, Sedaghat MM, et al. Autochthonous transmission of West Nile virus by a new vector in Iran, vector-host interaction modeling and virulence gene determinants. Viruses. 2020;12(12):1449. doi:10.3390/v12121449 33339336 PMC7766443

[25] Wongchitrat P, Chanmee T, Govitrapong P. Molecular mechanisms associated with neurodegeneration of neurotropic viral infection. Mol Neurobiol. 2024;61(5):2881–2903. doi:10.1007/s12035-023-03761-6 37946006 PMC11043213

[26] Linthout C, Martins AD, de Wit M, et al. The potential role of the Asian bush mosquito Aedes japonicus as spillover vector for West Nile virus in The Netherlands. Parasit Vectors. 2024;17(1):262. doi:10.1186/s13071-024-06279-5 38886805 PMC11181672

[27] Parker N. Exploring the role of temperature and other environmental factors in West Nile virus incidence and prediction in California counties from 2017-2022 using a zero-inflated model. PLoS Negl Trop Dis. 2024;18(6):e0012051. doi:10.1371/journal.pntd.0012051 38913741 PMC11226093

[28] Mor Z, Omari H, Indenbaum V, et al. Early rise of West Nile fever in Israel, June 2024. Euro Surveill. 2024;29(30):2400457. 39056196 10.2807/1560-7917.ES.2024.29.30.2400457 PMC11274845

[29] Marini G, Drakulovic MB, Jovanovic V, et al. Drivers and epidemiological patterns of West Nile virus in Serbia. Front Public Health. 2024;12:1429583. doi:10.3389/fpubh.2024.1429583 39086811 PMC11288825

[30] Magallanes S, Llorente F, Ruiz-Lopez MJ, et al. Warm winters are associated to more intense West Nile virus circulation in southern Spain. Emerg Microbes Infect. 2024;13(1):2348510. doi:10.1080/22221751.2024.2348510 38686545 PMC11073421

[31] Wang HR, Liu T, Gao X, et al. Impact of climate change on the global circulation of West Nile virus and adaptation responses: a scoping review. Infect Dis Poverty. 2024;13(1):38. doi:10.1186/s40249-024-01207-2 38790027 PMC11127377

[32] Tamura K, Stecher G, Kumar S. MEGA 11: molecular evolutionary genetics analysis version 11. Mol Biol Evol. 2021;38(7):3022–3027. 33892491 10.1093/molbev/msab120 PMC8233496

[33] Felsenstein J. Confidence limits on phylogenies: an approach using the bootstrap. Evolution. 1985;39(4):783–791. doi:10.2307/2408678 28561359

[34] Cvjetkovic IH, Radovanov J, Kovacevic G, et al. Diagnostic value of urine qRT-PCR for the diagnosis of West Nile virus neuroinvasive disease. Diagn Microbiol Infect Dis. 2023;107(1):115920. doi:10.1016/j.diagmicrobio.2023.115920 37390574

[35] Kasule S, Fernholz E, Grant L, et al. Whole-blood PCR preferred for timely diagnosis of neuroinvasive West Nile Virus infections: lessons from the 2021 Arizona outbreak. Open Forum Infect Dis. 2024;11(5):ofae188. doi:10.1093/ofid/ofae188 38680608 PMC11055396

[36] Girl P, Euringer K, Coroian M, et al. Comparison of five serological methods for the detection of West Nile Virus antibodies. Viruses. 2024;16(5):788. doi:10.3390/v16050788 38793670 PMC11126072

[37] Genc AC, Karabay O, Guclu E, et al. New prognostic parameter of West Nile Virus: platelet distribution width. Vector Borne Zoonotic Dis. 2024;24(3):166–171. doi:10.1089/vbz.2023.0056 37824783

[38] Khedhiri M, Chaouch M, Ayouni K, et al. Development and evaluation of an easy to use real-time reverse-transcription loop-mediated isothermal amplification assay for clinical diagnosis of West Nile virus. J Clin Virol. 2024;170:105633. doi:10.1016/j.jcv.2023.105633 38103483

[39] Garcia-Carrasco JM, Munoz AR, Olivero J, et al. West Nile virus in the Iberian Peninsula: using equine cases to identify high-risk areas for humans. Euro Surveill. 2023;28(40):2200844. doi:10.2807/1560-7917.ES.2023.28.40.2200844 37796440 PMC10557382

[40] Pelz JO, Muhlberg C, Friedrich I, et al. A specific pattern of routine cerebrospinal fluid parameters might help to identify cases of West Nile Virus neuroinvasive disease. Viruses. 2024;16(3):341. 38543707 10.3390/v16030341 PMC10974314

[41] Garcia-Zarandieta M, Quesada E, Martinez-Jimenez MI, et al. Identification of West Nile virus RNA-dependent RNA polymerase non-nucleoside inhibitors by real-time high throughput fluorescence screening. Antiviral Res. 2023;212:105568. doi:10.1016/j.antiviral.2023.105568 36842536

[42] Tang H, Liu Y, Ren R, et al. Identification of clinical candidates against West Nile virus by activity screening in vitro and effect evaluation in vivo. J Med Virol. 2022;94(10):4918–4925. doi:10.1002/jmv.27891 35644833

[43] Yang MJ, Luo HR, Fan ZY, et al. Development and evaluation of neutralizing antibodies for cross-protection against West Nile virus and Japanese encephalitis virus. Infect Med (Beijing). 2023;2(3):212–223. doi:10.1016/j.imj.2023.09.001 38073882 PMC10699678

[44] Liu YG, Peng HR, Ren RW, et al. CD11b maintains West Nile virus replication through modulation of immune response in human neuroblastoma cells. Virol J. 2024;21(1):158. doi:10.1186/s12985-024-02427-6 39004752 PMC11247799

[45] Flores HA, O'Neill SL. Controlling vector-borne diseases by releasing modified mosquitoes. Nat Rev Microbiol. 2018;16(8):508–518. doi:10.1038/s41579-018-0025-0 29777177 PMC7612058

[46] Kalmouni J, Will JB, Jr., Townsend J, et al. Temperature and time of host-seeking activity impact the efficacy of chemical control interventions targeting the West Nile virus vector, Culex tarsalis. PLoS Negl Trop Dis. 2024;18(8):e0012460. doi:10.1371/journal.pntd.0012460 39213461 PMC11392387

[47] Curren EJ, Shankar MB, Fischer M, et al. Cost-Effectiveness and impact of a targeted Age- and incidence-based West Nile Virus vaccine strategy. Clin Infect Dis. 2021;73(9):1565–1570. doi:10.1093/cid/ciab540 34117746 PMC9070563

[48] Naveed A, Eertink LG, Wang D, et al. Lessons learned from West Nile Virus infection:vaccinations in equines and their implications for One health approaches. Viruses. 2024;16(5):781. doi:10.3390/v16050781 38793662 PMC11125849

[49] Kaiser JA, Barrett ADT. Twenty years of progress toward West Nile Virus vaccine development. Viruses. 2019;11(9):823. doi:10.3390/v11090823 31491885 PMC6784102

[50] Bergmann F, Fischer D, Fischer L, et al. Vaccination of zoo birds against West Nile Virus-A field study. Vaccines (Basel). 2023;11(3):652. 36992236 10.3390/vaccines11030652 PMC10058624

[51] Habarugira G, Harrison JJ, Moran J, et al. A chimeric vaccine protects farmed saltwater crocodiles from West Nile virus-induced skin lesions. NPJ Vaccines. 2023;8(1):93. doi:10.1038/s41541-023-00688-w 37369653 PMC10300036

[52] Zarin DA, Tse T, Williams RJ, et al. The ClinicalTrials. gov results database—update and key issues. N Engl J Med. 2011;364(9):852–860. doi:10.1056/NEJMsa1012065 21366476 PMC3066456

[53] Hendy DA, Johnson-Weaver BT, Batty CJ, et al. Delivery of small molecule mast cell activators for West Nile Virus vaccination using acetalated dextran microparticles. Int J Pharm. 2023;634:122658. doi:10.1016/j.ijpharm.2023.122658 36731641 PMC9975031

[54] Karkashan A. Immunoinformatics assisted profiling of West Nile virus proteome to determine immunodominant epitopes for the development of next-generation multi-peptide vaccine. Front Immunol. 2024;15:1395870. doi:10.3389/fimmu.2024.1395870 38799422 PMC11116617

[55] Zhang YN, Li N, Zhang QY, et al. Rational design of West Nile virus vaccine through large replacement of 3’ UTR with internal poly(A). EMBO Mol Med. 2021;13(9):e14108. doi:10.15252/emmm.202114108 34351689 PMC8422072

[56] Abdel-Haleem Badr DR, Samy EE, Baker AM, et al. Larvicidal evaluation of two novel cationic gemini surfactants against the potential vector of West Nile virus Culex pipiens Linnaeus (Diptera: Culicidae). Med Vet Entomol. 2023;37(3):483–490. doi:10.1111/mve.12645 36799890

[57] Fulton CDM, Beasley DWC, Bente DA, Dineley KT. Long-term West Nile virus-induced neurological changes: A comparison of patients and rodent models. Brain Behav Immun Health. 2020;7:100105. doi:10.1016/j.bbih.2020.100105 34589866 PMC8474605

[58] Bakhiyi B, Irace-Cima A, Ludwig A, et al. Public health contributions of entomological surveillance of West Nile virus (WNV) and other mosquito-borne arboviruses in a context of climate change. Can Commun Dis Rep. 2024;50(9):394–304. doi:10.14745/ccdr.v50i09a02 PMC11383429 39257840

[59] Windah Tallei ALL, AlShehail TE, Suoth BM, et al. Immunoinformatics-driven strategies for advancing epitope-based vaccine design for West Nile Virus. J Pharm Sci. 2024;113(4):906–917. doi:10.1016/j.xphs.2023.11.025 38042341

